# Team building and leadership in the successful implementation of automation for high throughput screening

**DOI:** 10.1155/S1463924696000119

**Published:** 1996

**Authors:** Derek J. Hook

**Affiliations:** Bristol-Myers Squibb Biomolecular Screening 5 Research Parkway Wallingford CT 06492 USA


Journal of Automatic Chemistry, Vol. 18, No. 4 (July–August 1996), pp. 131–134

### Team building and leadership in the successful implementation of automation for high throughput screening

**Derek J. Hook**
*Bristol-Myers Squibb, Biomolecular Screening, 5 Research Parkway, Wallingford, CT 06492*

*This paper shows why high throughput screening programmes have become a fast growing area in which laboratory automation is playing a critical role. The unique philosophical and technical problems associated with high throughput screening are examined, and the paper explains how a team-based approach to solving these automation problems has become crucial in the successful implementation of these tasks.*

### Introduction

High throughput screening, perhaps more than any other laboratory automation task, illustrates the need for a diverse set of skills that can be brought together to solve a critical need in the pharmaceutical industry, namely the rapid and efficient discovery of new pharmacophores against a diverse set of disease targets. The resources needed for the development of an assay suitable for high throughput screening can include the scientific disciplines of molecular biology, biochemistry, analytical chemistry, computer database technology, robotics and automation, chemistry, industrial engineering, instrument development, statistical process control, project planning and electronics. In order to draw together the diverse set of talents that will be drawn on to get the screen into automated operation, managers need team-building and leadership qualities to achieve success. These leadership skills are needed to get commitment and buy-in, not only from the people implementing the screen on a day-to-day basis, but also those in the management of the research organization. This is necessary in order to sell the significant commitment in investment that modern state-of-the-art high throughput screening demands.

Also important is the involvement of the scientific staff and the management of the therapeutic areas for which the high throughput screen has been established, so that the lead compounds discovered during the screening process are evaluated properly in the follow-up assays for that disease, and become a source of lead chemotypes for synthetic chemical follow-up and target drug development.

The last two to three years have seen a massive increased interest in high throughput screening as a mechanism for new drug discovery. The reasons for this focus have come from the increased competitive pressures in the pharmaceutical market-place and show no signs of abating in the near future. The main competitive forces that are generating these pressures are from the changing health-care environment on costs and the demand for novel and effective therapies for chronic diseases. Even without government pressures in the USA for health care reform (cost containment), the market environment has changed considerably with the cost-containment pressures from Health Management Organizations (HMOs) and insurance companies on the pharmaceutical industry. Despite the high cost–benefit ratio that is realized from treating medical conditions with drugs, and the fact that they constitute only a small percentage of total health care costs, there is still considerable pressure for reducing the patient cost of pharmaceuticals. In addition, the selection of preferred drugs by HMOs, hospitals and government organizations for particular disease groups and the establishment of preferred formularies, has led to internal competitive pressures within the industry to be the first with novel therapies. This is to ensure that these products are on the preferred lists ahead of the competition.

The result of these developments has been the drive to produce new drug candidates from discovery research at an ever-increasing pace, with little or no prospect for massive increases in drug discovery resources to the research arm of the pharmaceutical industry.

Although high throughput screening has become a major factor in drug discovery for a balanced programme of drug discovery, a combination of approaches will yield the best results in the development of lead compounds. These approaches will include a combination of rational drug design based on knowledge of the three-dimensional structure of the target and active sites, traditional medicinal chemistry approaches based on chemical synthesis around known therapeutic agents or the best intuition as to potentially active compounds, and random high throughput screening.

However, the one advantage that high throughput screening in recent years has been able to offer is a relatively low cost to entry for the discovery of low molecular weight pharmacologic agents to many stellar biotechnology companies and it has received increased emphasis by the larger pharmaceutical houses as a mechanism for generating new drug candidates faster. The new biotechnology companies that have access to new targets through recombinant technologies have found that large molecular weight biologicals have a special set of development, drug delivery and approval problems that have not kept up with the promise of a few years ago. Small molecules are also a better source of drug candidates than expensive rational drug design programmes.

The promise that high throughput screening (HTS) offers pharmaceutical companies of any size is the potential to generate lead candidates quickly, and to potentially discover compounds with radically different pharmacophores than can be predicted on the basis of pre-existing

0142-0453/96 $12.00 © 1996 Taylor & Francis Ltd.

drugs or rational design criteria. These factors are now leading to the shortening of the discovery cycle and a movement away from 'metoo' compounds, but this pressure has resulted in new obstacles for high throughput screening. These are the numbers, chemical diversity of compound libraries, cost and time (how many compounds should be screened to discover with the highest probability desired, all potentially novel chemotypes, how diverse does a company's compound collection have to be to cover all possible pharmacophores for a given target; how fast can the library be screened; can this be done at a low enough cost to be economically feasible?). These questions are at the heart of state-of-the-art high throughput screening programmes, and have serious implications about how a successful HTS programme can be implemented and have a significant impact on the design of automated HTS systems.

### HTS today

Because of the increased competitive environment, the pressure to increase the sample throughput continues. In the late 1980s a general industry average for high throughput was about 75,000 compounds/screen per year to 18 months. A screen could run from a year to two years before being replaced by a new screen. Automation was minimal at best, simple robotic one arm systems. Compounds could be fed into the system within the screening department, and automated distribution systems were relatively unknown. Automation could be implemented either on a turn-key basis from a vendor (often with large system delivery times), or the system protocols could easily be established relatively slowly in house. Natural product screening was still an equal partner with synthetic chemical screening.

This scenario rapidly began to change in the early 1990s. The realization that a company's compound library was a potentially valuable screening source quickly led to the need to increase the pace at which compounds could be delivered to the screens existing in place at that time. This was coupled with the introduction of intelligent workstations, not only in the HTS laboratory, but in the research laboratories that simplified the liquid handling procedures needed for most biochemical and biological assays then used for high throughput screening. This resulted in more synthetic compound screening growing on an *ad hoc* basis, both in therapeutic area laboratories and HTS labs. Most pharmaceutical houses with compound libraries in the range of 75 000 to 150 000 compounds soon realized that some of the institutional barriers to synthetic compound screening had dropped, and that practical compound deck screening had arrived.

The infrastructure necessary to handle this process contributed to the move towards centralization of HTS departments. The realization that the discovery of a lead chemotype from a synthetic deck compound of known structure short-circuited the isolation/structure identification process from natural product leads has resulted in a vigorous debate about the contribution of natural product screening to drug discovery. This, in turn, has led to efforts to re-engineer the natural product drug discovery process to make it more efficient in the identification both of lead drug candidates from natural sources, and the identification of novel chemotypes that can be fed into the iterative cycle of structure-activity optimization. High throughput screening of both synthetic decks, and decks of commercially available natural product extracts, has become attractive to small biotechnology companies without the need to develop the costly, complex infrastructure necessary for fermentation and isolation of natural products.

The race to screen compound decks has begun in earnest and there are many more players than there were a decade ago.

What might have been 150 000 compounds in two years today approaches that same number in a short number of months. Without too much extrapolation into fantasy land, this period could easily become weeks or days. This compression of the cycle time for HTS has led to serious strains in the infrastructure supporting HTS, nowhere else more so than in the people developing, implementing and running the screens. This is where team building and leadership are so important to the successful implementation of the automation necessary to run samples through HTS screens at this rate.

As mentioned above, the reasons for the increased pressure to screen samples faster come from four forces that show no signs of abating in the near future and require a major shift in management's approach to both the personnel and capital infrastructure necessary for successful high throughput screening. These pressures come from health care reform; the human genome project (HUGO); the development of combinatorial chemistry; and the instrumental and technology advances generated by the microelectronics and miniaturization revolution. As these revolutionary developments continue to affect the pharmaceutical industry, management will need to turn more and more to finding ways to cope with the impact of these technologies on the work force. Automation, particularly, can be perceived as a threat to people's way of doing things, and the introduction of automation in an inappropriate way can lead to such resistance that the goals that are expected of automated systems are not achieved. One way to avoid this pitfall in implementing automation within HTS is to build in the commitment to automation as part of the team-building effort needed to achieve the goals of an HTS group.

The capital and skilled manpower requirements for HTS have led to the trend in most large pharmaceutical companies to develop and establish centralized HTS efforts. This minimizes the duplication of the capital investment in hardware, facilities and people. The last two Society for Biochemical Screening meetings have recognized that there is a growing specialization in skills for HTS, and that these skills are in short supply. The centralization of the HTS group does have dangers that have to be addressed by management. These include the need to get early interaction between the HTS group and therapeutic area scientists early to develop screens with assay protocols most suited to unattended, automated HTS; the development of recognition systems for those running the screens; and follow-up support and the education of upper management in the resource commitment for HTS. This not only includes the capital infrastructure necessary to screen, but the support for supply reagents to the screen, rapid follow up of leads biologically and sufficient chemistry support to develop lead chemotypes into drug candidates.

Infrastructure commitment not only means the capital necessary for the automated systems for screening, but provision of automated systems for the rapid follow-up of lead chemotypes, automated chemistry efforts for directed SAR synthesis, interaction with compound centers for automated storage, retrieval and dissolution systems, as well as the effective company-wide management of the data generated quickly and in large amounts from a good HTS effort. Management of HTS groups have to build these resources into a team that can respond quickly to both the results of a discovery effort, but also one that can quickly adapt to changing priorities for therapeutic area targets. Again it is clear that people issues, and getting them to function as a team leads to successful implementation of an HTS programme.

The trend towards the HTS as a service-oriented group to the different therapeutic areas also leads to problems in recognizing the real scientific contribution of the team that takes a new screen from cradle to grave, where the output is lead pharmacophores that often are altered beyond recognition in the drug candidate development process. The need for management and peers outside the HTS group to recognize these efforts as a real and tangible contribution to the drug discovery is often a real barrier to getting commitment of the HTS team from the start.

At Bristol-Myers Squibb, the involvement of the screening and therapeutic area scientists at the early stages of goal setting for implementation of a high throughput screen is considered vital to the successful achievement of that goal. This is illustrated by the company's experience with a rather cumbersome to implement enzyme screen that had become high priority in order to select a back-up candidate for a cardiovascular programme. It took the involvement of three people from the enzyme-based screening group and two people from the robotics group working as a team to develop novel product separation methodology that made the assay a viable one for HTS. It also took the involvement of therapeutic area scientists providing reagents and follow-up assays, and the commitment of one, and sometimes two, people from the compound distribution group to screen the entire compound deck within three to four months. A number of novel chemotypes emerged from this effort and it was successful because of the focus of the people involved working through many obstacles to implement the automation, resulting in the required throughput.

However, with the drive to increase throughput, the scope of automation becomes so great that even greater planning for the team's effort will start at earlier and earlier stages in the design of the assay for automation. This starts further and further back into the conceptualization phase of the target selection and assay design, and even greater team effort is required from both the therapeutic area scientists and earlier involvement of the various areas of expertise within HTS.

In addition, technology is driving change. Over the last few years, those assays that have the highest throughput and are easiest to develop, validate and implement are those that require the least actions on the part of automated systems. The best examples of these are the 'Mix and measure' type of assay. These can range from microbially based assays (both traditional antimicrobial assays, or assays based on engineering target genes into microbes with reporter systems), enzymic assays, or homogeneous assays (such as fluorescence polarization and the DELFIA time resolved fluorescence assays). Sportsman (Terrapin Technologies) has reported throughput of a microplate every three minutes using fluorescence polarization technology. This can result in over 400 microplates/day throughput with a sample capability of over 30 000 samples throughput per day. Assays that require complex separation steps (for example, radioligand binding assays with filtration steps) are assays that, while suitable for more modest throughput, are problematic with unattended automated systems.

It is important to recognize that, for successful application of automation, people skills are vital, and the technology needs are secondary. A manager with vision to lead the team consisting of individuals with a diverse set of skills is needed to inspire the team to success, and mould these individual skills towards a team goal rather than individual objectives. The leader also needs to recognize that the team does not function in a vacuum and has to share the vision of the goals, not only with the team, but with higher management who also have to commit resources in manpower and capital to the project.

In order to implement automation successfully, one must have a defined goal in mind for the outcome of the project. The procedure then has been broken down into its unit operations before automation is contemplated. This is the 'white board' stage of laboratory automation. If you outline the process and there is a step that you cannot do robotically, alarm bells should be sounding loud. This white board model also allows the critical team skills that the project needs to be pinpointed at the start, rather than half way through. At this stage, the team should meet and walk through the project, and responsibility should be accepted for the various steps. This may include biochemists to fine tune the chemistry or biology; the engineering support group to start to build all those little (or not so little) widgets that are needed; the data analysis people; and the automation group. At this time, the leader of the project should be selected and should begin to co-ordinate the process and build a project planning time line. Periodically, as the project progresses, assessments of progress and trouble-shooting sessions are necessary, until the preliminary implementation of the project is ready. Once the system is up and running for the first time, the same team members are needed for their feedback in validating the system, and also the follow-up during the initial running of the high throughput screen. Upper management should be kept informed of the progress of the project.

Following these guidelines will lead an automation project to success.

### Conclusion

No matter how much the people in a high throughput screening laboratory commit themselves to increased throughput by the use of appropriate automation, the pressure to develop the next generation of automation is always around the corner. The same competitive pressures that the pharmaceutical industry faces today will be present for the foreseeable future. This will be the result of the need for continued reduction in lead time to drug discovery, the continued impact of HUGO technology, the throughput 'forcing' by combinatorial and high speed parallel combinatorial synthetic chemistry and the renewed emphasis on improving the efficiency of natural products screening and the impact of new technologies. These include the recent proliferation of automation integrators and vendors (which will lead to an increase in the ease of use of automation, as each vendor tries to make the systems more modular, integrable and user-friendly). If we assume an analogous situation in automation and robotics to that which existed in the early days of analytical instrumentation, then the next few years should bring a low cost to automation and a more off-the-shelf approach to modular robotics systems. This approach to instrumentation will also impact on combinatorial chemistry. The ease of set up of combinatorial chemistry programmes using the off-the-shelf and easy-to-use instrumentation will continue the spiral of increased sample load into HTS systems. It will not be uncommon for compound collections to become 1 to 2 million in size. In addition, fall out of instrumentation from the HUGO project will affect the assay methodologies used for screen development. Microminiaturization of assays, including silicon-based chip sensors, micro-fabricated liquid-handling systems on a chip and modern sensor technology from the US defence programme will also impact the capability to screen large numbers of compounds cheaply. We are starting to see some of this technology already. The development of the 384-well microplate, whole plate CCD fluorescence readers, and newer 96-well pipetting devices are already in the market-place.

Also the impact of highly efficient, cheap computing power is making itself felt in harnessing the data torrent that is pouring out of HTS laboratories. Modern multitasking operating systems and object oriented programming techniques also promise to assist with the data flood. Artificial intelligence, neural networks and virtual reality techniques will all have some impact, the directions and applications of which we presently cannot conceive.

These developments will not let high throughput screening automation groups rest at today's level of throughput. The stage will be set for the conversion to full-scale production technology HTS for drug discovery. The company that seizes on this concept and this opportunity will be one of the few that will have the luxury of overflowing drug pipelines into the next century. Ultimately, however, it will not be the technology that will deliver this wealth, but the people behind the technology and their willingness to embrace change, function as a team to harness it, and to be the recipients of the rewards of that foresight.

In summary, people in HTS laboratories, and especially their management, face exciting challenges as we move into the next century. The industrial power inherent in the HTS philosophy promises to produce an abundance of new therapeutic agents, but can only be achieved by the total involvement of the HTS team in setting up and applying the technology necessary for any pharmaceutical company to be competitive in the market-place of today and the future.

